# Management of cardiovascular surgery in patients with systemic lupus erythematosus including thromboembolism and multiple organ failure prevention: A retrospective observational study

**DOI:** 10.1097/MD.0000000000032979

**Published:** 2023-02-17

**Authors:** Taira Yamamoto, Satoshi Matsushita, Daisuke Endo, Akie Shimada, Shizuyuki Dohi, Kan Kajimoto, Yasutaka Yokoyama, Yuichiro Sato, Yoichiro Machida, Tohru Asai, Atsushi Amano

**Affiliations:** a Department of Cardiovascular Surgery, Juntendo University Nerima Hospital, Tokyo, Japan; b Department of Cardiovascular Surgery, Juntendo University, Tokyo, Japan; c Department of Cardiovascular Surgery, Juntendo University Shizuoka Hospital, Shizuoka, Japan; d Department of Cardiovascular Surgery, Toda Chuo Hospital, Saitama, Japan.

**Keywords:** antithrombin III, cardiopulmonary bypass, cardiovascular surgery, systemic lupus erythematosus

## Abstract

Systemic lupus erythematosus is a chronic autoimmune disease that affects most tissues. Cardiovascular events are critical, life-threatening, long-term complications of systemic lupus erythematosus (SLE). We report our single-center experience of performing cardiovascular surgery in patients with SLE while avoiding postoperative complications. We also suggest a new approach for cardiopulmonary bypass and perioperative management. We applied the antiphospholipid antibody syndrome (APS) severity classification published by the Japan Intractable Disease Information Center to patients with SLE for perioperative management. Patients with Grade III or higher severity are treated with a slightly relaxed version of catastrophic APS therapy. This treatment modality includes glucocorticoids, anticoagulation, intravenous immunoglobulin, and plasma exchange. Between April 2010 and January 2021, 26 patients (2 males, 24 females) with SLE underwent cardiovascular surgery. The mean age was 74.2 ± 13.0 years (38–84 years). The primary outcomes were in-hospital mortality and long-term results, and the secondary outcomes were related to bleeding/embolization and coagulation function/platelet count. A subset analysis was performed to examine treatment efficacy in the APS Grade III or higher group. Of the 26 patients, 17 underwent valve surgery, 4 underwent isolated coronary artery bypass grafting, and 5 underwent thoracic aortic aneurysm surgery. There were no in-hospital deaths or associated bleeding/embolic complications. Postoperative antithrombin III decreased in patients who underwent valvular and aortic surgery, and platelet counts recovered to preoperative levels within 7 to 10 days. The 5- and 10-year survival rates were 80.5% and 53.7%, respectively. In addition, there were 10 patients with APS Grade III or higher, but there was no significant difference in the frequency of complications other than platelet recovery after treatment. The surgical outcome of open-heart surgery in patients with SLE was good. Surgical treatment of cardiovascular disease in these patients is difficult and complex. We focused on blood coagulation abnormalities and treated each patient by selecting the best individual treatment protocol according to the severity of the disease, taking into account the risk of bleeding and thrombosis. Management of blood coagulation function in these patients is essential, and careful therapeutic management should be considered during open-heart surgery.

## 1. Introduction

Systemic lupus erythematosus (SLE) is a chronic autoimmune disease that affects most tissues in the body. SLE is associated with significant complications, including infections, renal disease, cardiovascular diseases, and mortality.^[1]^ Although advances in medical management have dramatically improved the prognosis of SLE, early-onset SLE still presents with a higher frequency of severe clinical symptoms, recurrence, organ failure, and treatment side effects, as well as a longer duration of treatment.^[2]^ Cardiovascular complications are a significant factor in the later stages of disease development.^[3]^

According to the Japan Intractable Disease Information Center, there are approximately 60,000 patients with SLE in Japan. Approximately 40% of SLE patients are antiphospholipid antibody positive, and about 10% to 20% develop antiphospholipid antibody syndrome (APS). Of these, it has been reported that <40% develop thrombosis.^[4]^

APS is an autoimmune disease caused by the presence of antiphospholipid antibodies such as lupus anticoagulant (LAC), anticardiolipin antibodies, or anti-β2 glycoprotein-I antibodies, thus resulting in recurrent arteriovenous thrombosis and failure to thrive. When left untreated, arterial and venous thrombosis has been reported to recur in 50% of cases within 6 months. In APS patients, infections and surgery can lead to catastrophic APS (CAPS).^[5]^

In cardiovascular surgery for patients with SLE, it is essential to have a management strategy to prevent perioperative bleeding and embolism. Generally, a low platelet count, low platelet function, and ATIII consumption are significantly associated with bleeding after open-heart surgery.^[6,7]^ However, to the best of our knowledge, there are few publications on the development and modification of surgical techniques in patients with SLE, and no studies on postoperative platelet count recovery, bleeding, or embolic complications based on preoperative clinical and laboratory findings.

This study examined the perioperative management, postoperative results, and long-term outcomes of cardiovascular surgery performed in our hospital for ischemic heart disease, valvular disease, and aortic disease in patients with SLE.

## 2. Methods

### 2.1. Patients

A retrospective observational study was performed on the perioperative and remote outcomes of patients with SLE who underwent cardiovascular surgery. We performed cardiovascular surgery on 26 patients (2 males and 24 females) with SLE from April 2010 to January 2021. The mean ± standard deviation age was 56 ± 13 years (range, 38–84 years). Of the 26 patients, 17 underwent valvular surgery, 4 underwent isolated coronary artery bypass grafting (CABG), and 5 underwent thoracic aortic aneurysm surgery (Table 1).

**Table 1:** Severity classification of antiphospholipid antibodies.

Grade	Definition
0	Not applicable to APS
I	No treatment required, no organ damage, and no decline in ADL. 1) No antiplatelet or anticoagulation therapy and no new onset of thrombosis within the past year. 2) A history of pregnancy complications only, without a history of thrombosis. 3) There is a history of thrombosis but no evidence of organ damage and no interference with daily life.
II	The patient is treated but stable, with no organ damage and no ADL decline.
	The patient is on antiplatelet or anticoagulation therapy and has no new onset of thrombosis within the past year; there is a history of thrombosis but no organ damage and no interference with daily life.
III	Recurrent thrombosis despite treatment, mild organ damage, and decreased ADL. 1) Recurrent thrombosis: New thrombosis within the past year despite antiplatelet or anticoagulation therapy. 2) Mild organ damage: permanent organ damage due to APS (cerebral infarction, myocardial infarction, pulmonary infarction, renal damage, vision loss, or visual field abnormality, among others) but little or no decline in ADL.
IV	During treatment for antiphospholipid antibody-related diseases, during pregnancy management, with moderate organ damage or decline in ADL. 1) Antiphospholipid antibody-related disease: In addition to APS with a confirmed diagnosis, ongoing immunosuppressive therapy for antiphospholipid antibody-related thrombocytopenia, neuropathy. 2) Pregnancy management: she had antiplatelet therapy or anticoagulation therapy within the past year to prevent thrombosis during pregnancy or pregnancy complications. 3) Moderate organ damage: there is permanent vital organ damage (cerebral infarction, myocardial infarction, pulmonary infarction, renal impairment, vision loss, or visual field abnormality, among others) due to APS and ADL being decreased.
V	Catastrophic APS, new or relapse of antiphospholipid antibody-related disease requiring treatment, pregnancy complications during therapy, severe organ damage, and decreased ADL. 1) CAPS: onset within the past year, requiring multidisciplinary treatment. 2) Antiphospholipid antibody-related disease: In addition to APS with a confirmed diagnosis, immunosuppressive therapy for antiphospholipid antibody-related thrombocytopenia, neuropathy has been initiated within the past year, or treatment has been intensified due to relapse. 3) Severe organ damage: significant loss of ADL such as the need for assistance due to permanent vital organ damage caused by APS (cerebral infarction, myocardial infarction, pulmonary infarction, renal impairment, vision loss, or visual field abnormality).

ADL = activity of daily life, APS = antiphospholipid antibody syndrome, CAPS = catastrophic antiphospholipid syndrome.

### 2.2. Surgical procedure

#### 1.2.2. Coronary artery bypass grafting.

**Figure 1.**
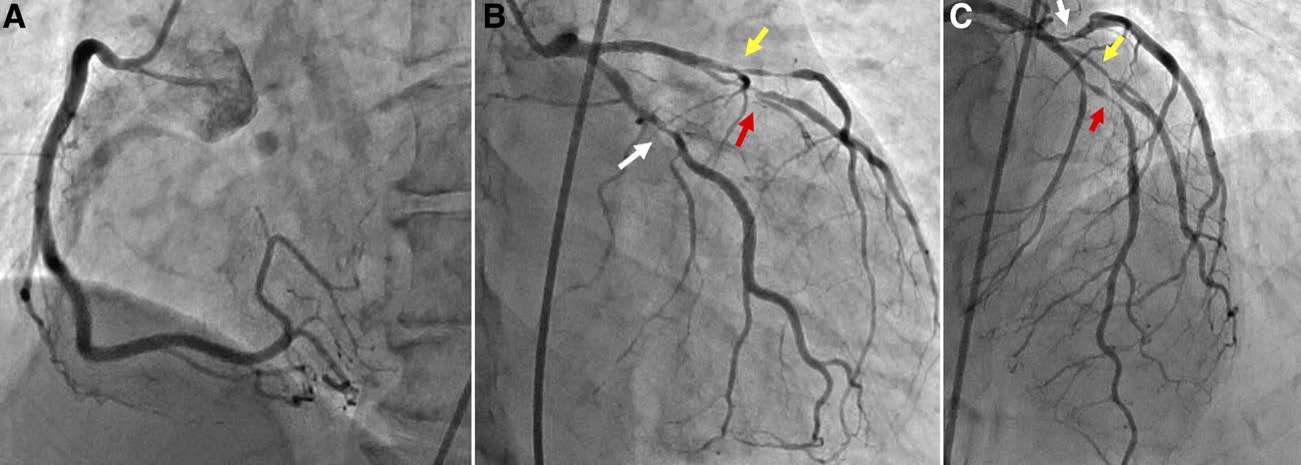
Coronary artery disease. The patient was a 36-year-old woman with a 23-year history of systemic lupus erythematosus and triple factor-positive antiphospholipid antibody syndrome. She is currently in the negative phase of the disease, following daily oral prednisolone 9 mg and immunosuppressive therapy. (A) Preoperative CAG showed a normal right coronary artery. (B and C) Preoperative CAG was used to visualize the 95% stenosis in the middle left anterior descending artery (red arrow), 95% stenosis at the first diagonal branch (yellow arrow), and 50% stenosis in the distal left circumflex artery (white arrow). CAG = cineangiography.

**Figure 2.**
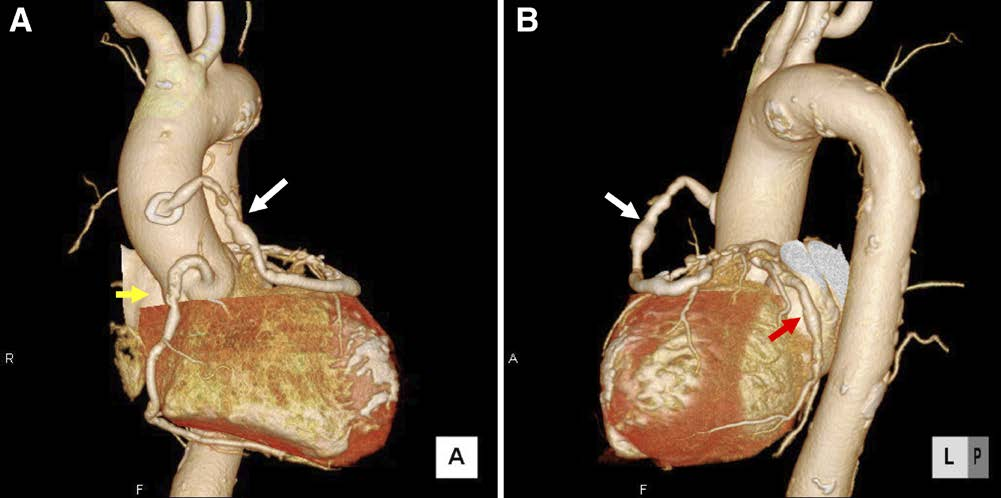
Coronary aneurysms and stenosis. Saphenous vein disease in prior CABG. The patient was a 59-year-old woman with a 10-year history of SLE and a history of CABG surgery 16 years earlier. She was treated with 5 mg of prednisolone daily, and the SLE was stable. ECG-gated 3D-CT was used to visualize the diseased saphenous vein graft (white arrow), dilated right coronary artery (yellow arrow, A), and dilated left circumflex artery (red arrow, B). 3D-CT = three-dimensional computed tomography, CABG = coronary artery bypass grafting, ECG = electrocardiography, SLE = systemic lupus erythematosus.

We performed isolated off-pump CABG (OPCAB) to prevent bleeding and thrombosis complications associated with cardiopulmonary bypass (CPB). We also prioritized the use of arterial grafts for patients with SLE as often as possible owing to the vasculitis and thrombogenic potential in SLE. In SLE, coronary artery stenosis often develops at a young age; therefore, CABG is given first preference over interventional coronary stenting (Fig. 1A–C). In 1 patient who previously underwent CABG using a saphenous vein graft, we performed CABG using 3 arterial grafts with 5 anastomoses (Fig. 2A and B). We did not discontinue aspirin (100 mg/d) in the perioperative period and used heparin during OPCAB to control the activated clotting time (ACT) to be ≥300 seconds.

#### 2.2.2. Valvular surgery.

**Figure 3.**
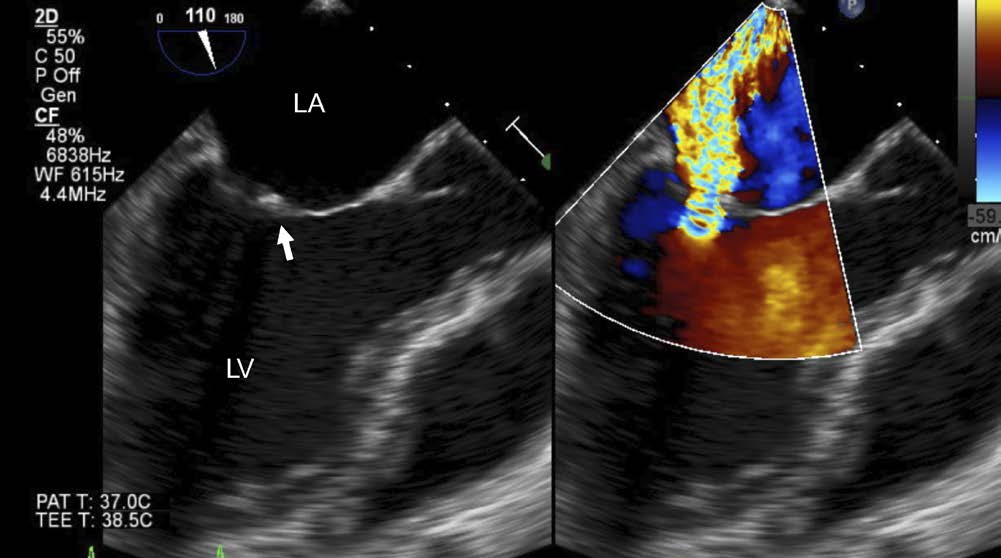
Severe mitral regurgitation in a patient with systemic lupus erythematosus with non-bacterial endocarditis. The patient was a 42-year-old woman with SLE and double-factor positive antiphospholipid antibody syndrome. Mitral regurgitation and endocarditis were suspected, and she was admitted to the hospital as an emergency. TEE showed severe mitral regurgitation and non-bacterial verrucous vegetations at the anterior leaflet (white arrow). LA = left atrium, LV = left ventricle, SLE = systemic lupus erythematosus, TEE = transesophageal echocardiography.

**Figure 4.**
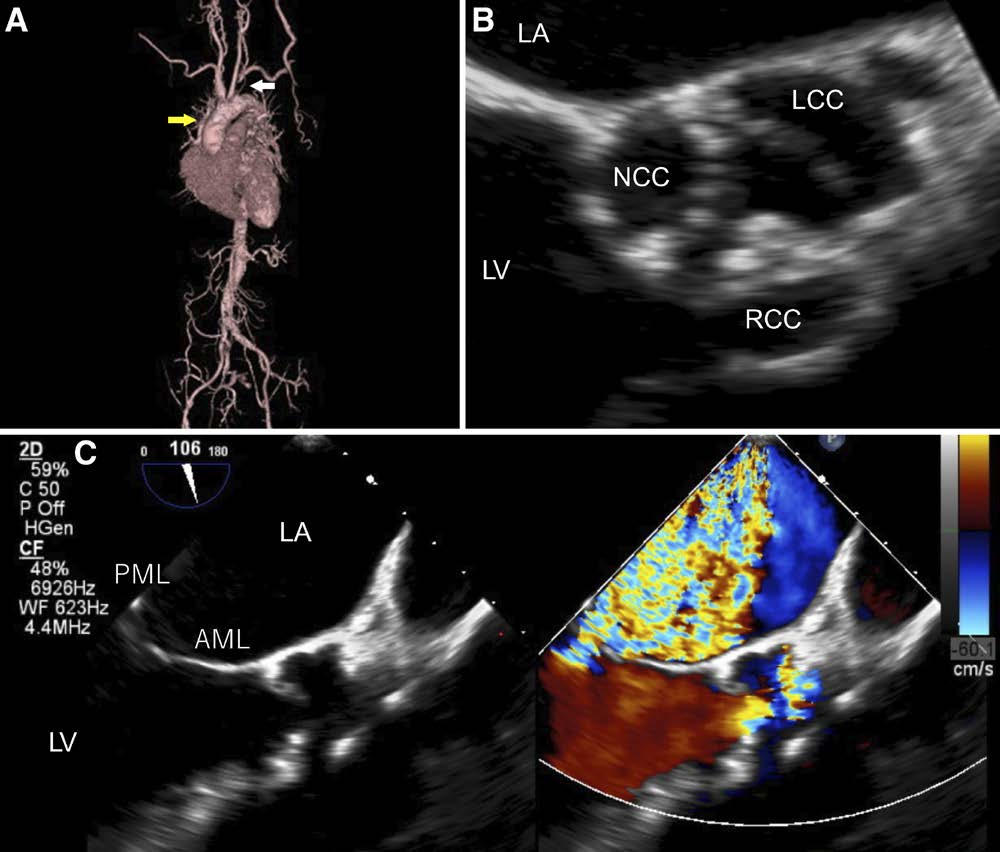
Severe aortic valve stenosis and mitral regurgitation in a patient with systemic lupus erythematosus on hemodialysis due to lupus nephritis. The patient was a 59-year-old woman with a 15-year history of SLE. She was introduced to dialysis 10 years earlier due to lupus nephritis. She also had an IVC filter implanted for deep vein thrombosis. (A) 3D-CT shows multiple atheromas around the thoracic aorta and occlusion in the left subclavian artery (white arrow). The ST junction is smaller than the annulus, and the gap is recognized below the commissure. (B and C) TEE showed severe aortic stenosis and mitral regurgitation. The opening of the RCC and NCC of the aortic valve is severely restricted due to severe calcification, and there is mitral regurgitation due to severe calcification of the posterior leaflet of the mitral valve. 3D-CT = three-dimensional computed tomography, AML = anterior mitral leaflet, IVC = inferior vena cava, LA = left atrium, LV = left ventricle, LCC = left coronary cusp, NCC = non-coronary cusp, PML = posterior mitral leaflet, RCC = right coronary cusp, SLE = systemic lupus erythematosus, TEE = transesophageal echocardiography.

We performed valvular surgery in 17 patients and mitral valve surgery in 10 patients. We performed mitral valvuloplasty in 7 patients and mitral valve replacement in 3 patients (1 patient had mitral stenosis and 2 patients had infective endocarditis). In addition, aortic valve surgery was performed in 9 patients (aortic valve replacement in six, aortic root reconstruction in two, and aortic valvuloplasty in one). In mitral valve surgery, mitral valvuloplasty was the first choice, and we decided to perform mitral valve replacement in cases of severe mitral valve destruction. There were variations in the cases of infective and aseptic endocarditis, and we opted for mitral valvuloplasty following a thorough examination of SLE and APS (Fig. 3). Considering aortic valve pathology, 7 patients had aortic stenosis and three had aortic regurgitation. We opted for mechanical valves, except in patients aged > 70 years. Dialysis might be inducted at a relatively young age in patients who have been treated for a long time for SLE or who have advanced Lupus nephritis. In such cases, atherosclerosis of the aorta progresses, and calcification of both aortic and mitral valves becomes severe (Fig. 4A–C).

#### 3.2.2. Thoracic aortic surgery.

**Figure 5.**
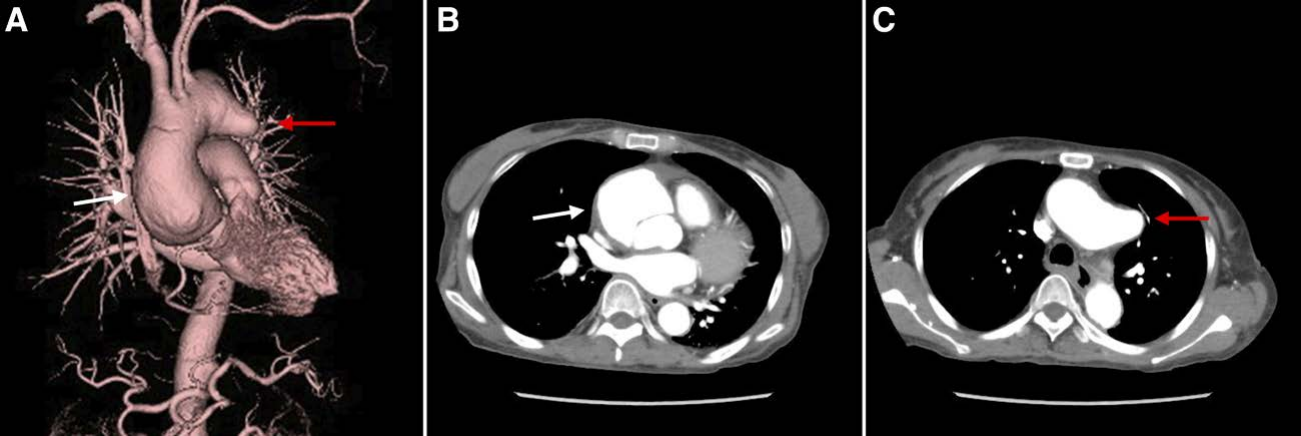
Acute aortic dissection (DeBakey type II) and true saccular aneurysm. The patient was a 55-year-old woman with a 33-year history of SLE. She was taking 15 mg of prednisolone daily. She also had an IVC filter implanted for deep vein thrombosis. 3D-CT showed aortic dissection (DeBakey type II, white arrow) and a true saccular aneurysm at the distal arch (red arrow). 3D-CT = three-dimensional computed tomography, IVC = inferior vena cava, SLE = systemic lupus erythematosus.

We performed thoracic aortic replacement in 5 patients. Two patients had type II acute aortic dissection and three had true aortic aneurysms (Fig. 5A–C). Aortic aneurysm and dissecting aortic aneurysm cases are very diverse; they include the elderly, long periods of SLE, or dialysis (lupus nephritis). Therefore, perioperative treatment management, including CPB, is critical.

### 2.3. Anticoagulation management during CPB

Similar to conventional cardiovascular surgery, the patient was placed on CPB, valve surgery was performed during cardiac arrest, and thoracic aortic aneurysm surgery was performed using the hypothermic circulatory arrest (selective cerebral isolated perfusion) method. The hypothermic circulatory arrest method was set at a rectal temperature of 28°C.

It is widely believed that a heparin concentration of 3 U/mL in the blood during CPB is sufficient for adequate anticoagulation^[8]^; therefore, an ACT of ≥ 450 seconds, which corresponds to that value, is considered ideal.^[9]^ However, excessive heparin administration is thought to result in postoperative heparin rebound.^[10]^ Therefore, at our institution, we calculate and administer the dose of heparin required for the target ACT based on the ACT measured at the start of the surgery. The heparin dose was calculated based on the patient’s body weight (300 IU of heparin per kg). As previously described,^[9]^ the target ACT was 450 seconds. Based on a normal ACT value of 100 seconds, the administered dose was corrected according to the ACT. Three hundred units of heparin per kilogram of body weight was adjusted by the baseline ACT (bACT): body weight (kg) × 300 IU × (450 − bACT)/(450 − 100).^[11]^

We measure the platelet antibody titer in the presence of clinically persistent thrombocytopenia. When heparin-induced thrombocytopenia (HIT) is strongly suspected, we use latex immunoturbidimetry (functionalized immunoassay) as an immunoassay to detect HIT antibodies. If this test is positive, a heparin-induced platelet aggregation test is additionally performed as a functional assay, and if both tests are positive, HIT is diagnosed.

The Practice Guidelines-Anticoagulation During Cardiopulmonary Bypass by the Society of Thoracic Surgeons, Society of Cardiovascular Anesthesiologists, and American Society of In Vitro Technologies recommend the anticoagulant antithrombin bivalirudin as an anticoagulant for patients who cannot use heparin, while argatroban, which frequently causes bleeding complications, is not recommended.^[12]^ However, bivalirudin is not yet an approved drug in Japan. Therefore, argatroban has been used in many facilities in Japan, although its use is highly complicated. There is no consensus on the indications and dosage of argatroban during CPB; furthermore, the dosage is controlled by ACT, which is often prolonged for several hours longer than expected, even after the completion of CPB. The half-life of argatroban is 42 minutes; however, it has been reported to be prolonged approximately 10-fold when using CPB.^[13]^ In cases of HIT, we used nafamostat mesylate for anticoagulation during CPB. Nafamostat mesylate has a short half-life and is easy to use to control ACT; however, it is a very expensive drug.^[14]^ Anticoagulation during CPB was managed as follows. Nafamostat mesylate was administered through the venous circuit line in the range of 0 to 2.0 mg/kg/h just before the passage of the artificial lung. Heparin was initially used in small doses (100 U/kg), and nafamostat mesylate at 2.5 mg/kg/h was started after the initial ACT of 250 to 275 seconds was confirmed. After the first time, no heparin was used. Continuous dosing was stopped at 1.0 mg/kg/h between 250 and 300 seconds, 0.5 mg/kg/h between 300 and 350 seconds, 0.25 mg/kg/h between 350 and 400 seconds, 0.125 mg/kg/h between 400 and 450 seconds, and at ≥450 seconds. We measured the ACT from the venous circuit line maintained between 250 and 450 seconds during CPB; approximately 10 minutes before the end of CPB, we stopped the continuous injection of nafamostat mesylate and have not used protamine at all.

### 2.4. Management according to APS severity

Patients with SLE may have no noticeable clinical signs of APS preoperatively; however, after surgery, complications may lead to more pronounced APS or, in the worst case, CAPS. In 2 previous reports of distant outcomes, approximately 30% of APS cases were complicated by SLE; however, the 10-year survival rate was >90%. However, many cases are refractory to treatment, and the importance of prognostic markers and appropriate treatment is stated.^[15–17]^ The severity classification of APS presented by the Japan Intractable Disease Information Center is shown in Table 1.^[18]^ We also calculated another classification, the Global Anti-Phospholipid Syndrome Score.^[19]^

CAPS is a group of antiphospholipid antibody-related diseases requiring new or relapsed treatment and targets the following cases. If it develops within the past year and required multidisciplinary treatment. Pregnancy complications during treatment within 1 year related to APS. In cases that require immunosuppressive therapy for APS-related thrombocytopenia and neuropathy if started or resumed within a year. Cases with severe permanent, substantial organ damage or decreased activities of daily living associated with APS.^[20]^

In our department, we use the CAPS protocol in the perioperative period for Grade III and higher APS cases, as we believe that the risk is equivalent to CAPS.^[4]^ We administered a continuous heparin infusion (10,000–15,000 units/24 h) 5 days before surgery. In these cases of SLE with APS, it is essential to know the accurate heparin concentration; however, it takes several days to obtain the test results. Therefore, in practice, the ACT and activated partial thromboplastin time are measured in SLE cases, and we always caution against ACT measurement errors. We also administered intravenous gamma globulin (IVIG, 200 mg/kg/d) for 2 days before, on the day of, and 2 days after the surgery. During cardiopulmonary ventilation, we performed plasma exchange. Steroid pulses of 1000 mg methylprednisolone sodium succinate were administered on the day of surgery and the first postoperative day. If the postoperative platelet count decreased to <40,000 and fibrin degradation products increased even on the third postoperative day, IVIG was administered for another 3 days after surgery. Methylprednisolone doses were determined empirically on a case-by-case basis. The decision to perform IVIG at the same time was also based on empirical judgment.

### 2.5. Data collection

All 26 consecutive patients underwent successful cardiovascular surgery between April 2010 and January 2021 in the Department of Cardiovascular Surgery, Juntendo University Hospital. Written consent was obtained from all patients for the publication of the manuscript. The Clinical Ethics Committee of Juntendo University Hospital approved this study, and the study was conducted in accordance with the principles of the Declaration of Helsinki. We worked with collagenologists, cardiologists, and nephrologists to provide perioperative SLE medical management. The data collected included the preoperative treatment history, preoperative and postoperative general condition, medical treatment, biochemical tests, and hemodynamic valve performance on transthoracic echocardiography and electrocardiography-gated 3-dimensional computed tomography. Data regarding the long-term prognosis were obtained from the medical records as all the patients were being attended to in our internal medicine department and our department.

The primary outcomes were hospital mortality, morbidity, and long-term follow-up results. We analyzed secondary outcomes, especially those related to bleeding/embolization and coagulation function/platelet counts. In addition, we performed a subset analysis to examine the relationship between outcomes and severe cases of APS above Grade III.

### 2.6. Statistical analyses

We assessed the normality of the distribution of the continuous variables using the D’Agostino-Pearson analysis. We used descriptive statistical methods to depict the study population, and continuous variables are shown as means and standard deviations. We also report the total number and proportions of categorical outcomes. Moreover, we have presented the numbers and percentages of actual surgical outcomes as initial events, as well as deaths and readmissions during the long-term follow-up. Continuous variables were compared using a *t* test or Wilcoxon or Mann–Whitney *U* tests (non-parametric variables). Categorical variables were compared using Pearson’s v2 test. A 2-tailed *P* value of <.05 was considered significant. All data were analyzed using JMP software, version 16.0 (SAS Institute, Cary, NC).

## 3. Results

### 3.1. Demographics and baseline characteristics

Between April 2010 and January 2021, cardiovascular surgery was performed on 26 patients with SLE at our institution. This included 4 cases of isolated CABG, 17 cases of valvular surgery, and 5 cases of aortic surgery. The mean ages of the patients who underwent CABG, valvular surgery, and aortic surgery were 50, 54, and 67 years, respectively. Except for the 2 cases of valvular surgery, all the patients were female. Hypertension, diabetes mellitus, impaired renal function, and previous stroke were more prevalent in patients who underwent aortic surgery. The aortic surgery group had a greater number of older patients. However, dyslipidemia was more common in patients with coronary artery disease. The EuroScore II score, a risk analysis model that predicts postoperative mortality after open-heart surgery, was higher in patients with aortic disease, and these patients had more comorbidities (Table 2).

**Table 2:** Preoperative patient baseline characteristics.

	Isolated CABG	Valvular surgery	Aortic surgery
Number of patients	4	17	5
Age (yr)	50 ± 16 (34–67)	54 ± 12 (41–72)	67 ± 11 (55–81)
Female sex	4 (100%)	15 (88%)	5 (100%)
Body mass index (kg/m^2^)	21.42 ± 3.66	20.87 ± 2.71	18.40 ± 2.02
Body surface area (m^2^)	1.49 ± 0.09	1.51 ± 0.17	1.34 ± 0.09
Brain natriuretic peptide (pg/mL)	74 ± 95	722 ± 2010	1667 ± 3540
Diabetes mellitus	0	2 (11.8%)	3 (60.0%)
Hypertension	2 (50.0%)	8 (47.1%)	4 (80.0%)
Hyperlipidemia	3 (75.0%)	6 (35.3%)	1 (20.0%)
Cerebrovascular accident	1 (25.0%)	4 (23.5%)	2 (40.0%)
Estimated glomerular filtration (rate mL/min/1.73 m^2^)	72.5 ± 37.0	56.5 ± 31.8	46.9 ± 26.5
Hemodialysis (Lupus nephritis)	0	3 (17.7%)	1 (20.0%)
Chronic lung disease	0	1 (5.9%)	0
Malignancy	1 (25.0%)	2 (11.8%)	1 (20.0%)
Peripheral vascular disease	0	1 (5.9%)	0
Previous cardiovascular interventions	0	0	0
Coronary artery disease	4 (100%)	3 (17.7%)	2 (40%)
Number of diseased vessels 1VD	0	1	1
2VD	2	1	0
3VD	2	1	1
Previously PCI	0	1 (5.9%)	0
Previous cardiac surgery	1 (25.0%)	0	0
Previous pacemaker implantation	0	0	0
Previous atrial fibrillation	0	1 (5.9%)	0
Euro Score II	2.23 ± 2.80	2.64 ± 1.74	15.0 ± 19.0

1VD = one vessel disease, 2VD = two vessels disease, 3VD = three vessels disease, CABG = coronary artery bypass surgery, EuroSCORE = European system for cardiac operative risk evaluation, PCI = percutaneous coronary intervention.

All the patients, except one who underwent aortic surgery, were treated with prednisolone or immunosuppressive drugs, and their SLE activity was stable. A history of miscarriage was found in 3 cases of aortic disease; however, we were unable to obtain the relevant details. Furthermore, 30% to 40% of patients with valvular and aortic surgery had a history of deep vein thrombosis. Approximately 20% to 40% of the patients had a history of cerebral thromboembolism. Six patients were still on anticoagulation (five on warfarin, one on a direct oral anticoagulant), and the target value of the prothrombin time-international normalized ratio was 2.0 to 2.5. Antinuclear antibodies were high; however, immunoglobulins and complement did not decrease, and the immune status was stable (Table 3).

**Table 3:** Preoperative baseline characteristics of patients with SLE.

	Isolated CABG	Valvular surgery	Aortic surgery
Duration of SLE (yr)	19.5 ± 7.9	20.9 ± 14.9	24.4 ± 14.7
Other collagen disease	2 (50.0%)	2 (12.5%)	1 (25.0%)
Treatment with PSL	4 (100.0%)	14 (82.4%)	4 (80.0%)
PSL ≥ 10 mg	1 (25.0%)	5 (29.4%)	2 (40.0%)
PSL < 10 mg, ≥5 mg	3 (75.0%)	7 (41.2%)	2 (40.0%)
PSL < 5 mg	0	5 (29.4%)	1 (20.0%)
Immunosuppressants	2 (50.0%)	5 (29.4%)	0
Platelet (×104/μL)	19.2 ± 3.2	18.4 ± 7.6	17.2 ± 8.7
D-dimer (μg/mL)	1.2 ± 0.2	8.8 ± 23.6	38.0 ± 39.0
Antithrombin III (%)	115 ± 14	104 ± 16	97 ± 14
Past history of miscarriage	0	0	3 (60.0%)
Early pregnancy loss (<10 wk)	0	0	3
Live births			
History of stroke, infarction	1 (25.0%)	4 (23.5%)	2 (40.0%)
History of deep vein thrombi	0	5 (29.4%)	2 (40.0%)
Pulmonary embolism	0	0	0
Catastrophic APS	0	0	0
Others thrombosis	0	0	0
Immunoglobulin G (mg/dL)	1186 ± 24	1263 ± 553	1260 ± 553
C3 (mg/dL)	91.0 ± 13.4	80.2 ± 15.2	98.6 ± 17.1
C4 (mg/dL)	17.3 ± 3.4	19.6 ± 10.0	20.2 ± 6.4
CH50 (U/mL)	40.8 ± 9.6	35.9 ± 7.7	42.3 ± 9.4
Anti-nuclear antibody	120 ± 134	252 ± 622	240 ± 622
APS	2 (50.0%)	8 (47.1%)	2 (40.0%)
LAC positivity	0	4 (23.5%)	1 (20.0%)
aCL antibody positivity	2 (50.0%)	2 (11.8%)	0
CLB2GP1 positivity	1 (25.0%)	5 (29.4%)	0
Triple positivity	0	0	0
Double positivity	1 (25.0%)	3 (17.7%)	1 (20.0%)
GAPPS > 16	0	0	0
Severity grade of APS			
0	1	1	3
I	0	1	1
II	2	0	7
III	0	1	3
IV	1	1	3
V	0	1	0
Preoperative anticoagulation therapy	1 (25.0%)	3 (17.7%)	2 (40.0%)
Preoperative heparin infusion	3 (75.0%)	10 (58.8%)	4 (80.0%)
Preoperative IVIG	2 (50.0%)	9 (52.9%)	3 (60.0%)
Heparin-induced thrombocytopenia	0	1 (5.8%)	1 (20.0%)

aCL positivity was defined as ≥ 10 U/mL.

LAC positivity was defined as > 1.2 U.

CLB2GP1 positivity was defined as ≥3.5 (U/mL).

The factor indicates the number of positive factors for each case (aCL, LAC, and CLB2GP1).

aCL = anti-cardiolipin, APS = antiphospholipid antibody syndrome, CABG = coronary artery bypass grafting, CLB2GP1 = anticardiolipin antibody, cardiolipin antibodyβ2-glycoprotein-1 complex, GAPPS = Global Anti-Phospholipid Syndrome Score, IVIG = intravenous gamma globulin, LAC = lupus anticoagulant, PSL = prednisolone.

We used a phospholipid-dependent coagulation test called LAC, antibodies against the cardiolipin-beta-2-glycoprotein-1 complex, and anticardiolipin antibodies to determine the positivity rates of these 3 factors. Fortunately, there were no cases of triple-positivity; however, double-positivity, in which 2 items were positive, was observed in approximately 20% of the cases. Platelet counts were maintained; however, D-dimer levels increased in valvular and aortic cases, and antithrombin III levels decreased. We infused heparin preoperatively in 50% to 80% of cases and IVIG in 50% to 60% of cases. Two cases of HIT were observed, and the platelet counts ranged from 6.0 × 10^4^ to 10.0 × 10^4^/μL (Table 3).

The left ventricular ejection fraction tended to be lower in CABG and higher in aortic surgery. The diameters of the aortic valve leaflets, Valsalva, and ascending aorta were not significant in patients with CABG and valvular surgery. Mitral valve disease and aortic valve disease mainly included mitral regurgitation and aortic stenosis, respectively. All 5 cases of thoracic aortic disease revealed dilatation of the ascending aorta, and 2 cases revealed aortic dissection. One patient had a saccular aneurysm in the distal aortic arch (Table S1, Supplemental Digital Content, http://links.lww.com/MD/I490).

### 3.2. Clinical outcomes

We performed emergency surgery in 2 cases of suspected infective endocarditis and 1 case of acute aortic dissection. We performed CABG using OPCAB, except for 1 case of reoperation. In mitral valves, we performed mitral valvuloplasty, and mitral valve replacement was performed using a mechanical valve in cases of regurgitation or stenosis owing to severe mitral valve degeneration or calcification. Subsequent anticoagulation therapy was continued in 25% to 50% of cases (Table S2, Supplemental Digital Content, http://links.lww.com/MD/I491).

The surgical results of our strategy were excellent, and the patients were completely controlled, especially with regard to hemorrhage and thromboembolism, which are of particular concern. There was only 1 case of superficial incisional infection and 2 cases of respiratory complications, but no pneumonia. Continuous hemodiafiltration was warranted in 3 cases. However, these patients did not progress to chronic renal failure. The average length of hospital stay was 13 days for OPCAB without cardiopulmonary resuscitation and 30 days for aortic surgery with hypothermic circulatory arrest (Table S2, Supplemental Digital Content, http://links.lww.com/MD/I491).

The blood transfusion volume was increased due to the degree of anemia and coagulation function. Antithrombin III at the time of return to the ICU after surgery was decreased in patients using CPB, whereas it was maintained in OPCAB. Platelet counts recovered to preoperative levels within 5 to 7 days following OPCAB; however, 7 to 10 days were required after valvular and aortic surgery (Table S3, Supplemental Digital Content, http://links.lww.com/MD/I492).

Although we performed exceptional perioperative management for the 10 patients with Grade III or higher, the results concerning postoperative complications were similar to those for Grades I to II. Even with these intensive treatments, the platelet count recovery postoperatively was delayed in the Grade III and higher group (Table S4, Supplemental Digital Content, http://links.lww.com/MD/I493).

**Figure 6.**
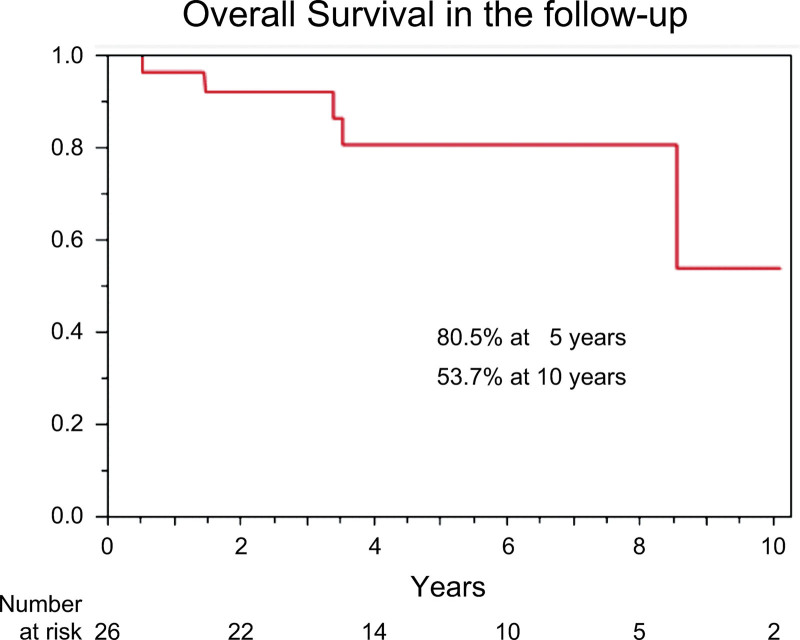
Overall survival (follow-up). The Kaplan–Meier curve, showing overall survival with 5- and 10-year overall survival rates of 80.5% and 53.7%, respectively.

The overall survival Kaplan–Meier curve is shown in Figure 6. The 5- and 10-year overall survival rates were 80.5% and 53.7%, respectively. The results were satisfactory considering the length of time the patients had been living with SLE at the time of surgery. SLE patients who died during follow-up had a disease-related period of 23 to 43 years (Table 4). Patients were more likely to be hospitalized for long-term follow-up due to systemic complications associated with SLE, independent of surgery. These events occurred regardless of the severity of APS due to the progression of atherosclerosis associated with SLE (Table 5).

**Table 4:** Mortality during follow-up.

Patient no.	Age at operation (yr)	Age at death	Cause of death	Surgical procedure	Duration of SLE (yr)
1	65	69	Gallbladder cancer	AVR	35
2	52	60	Pneumonia	MVR TAP	23
3	75	78	Ischemic colitis	TAR	32
4	70	71	AMI	AVR MVP	38
5	83	85	Pneumonia	AVR TAP	43

AMI = acute myocardial infarction, AVR = aortic valve replacement, MVP = mitral valve plasty, MVR = mitral valve replacement, SLE = systemic lupus erythematosus, TAP = tricuspid annular plasty, TAR = total arch replacement.

**Table 5:** Comorbidities according to the severity grade of antiphospholipid antibody syndrome during follow-up.

Grade at the operation	Number of patients	Mortality	Number of patients with any event during follow up	Age	Type of operation	Type of event (follow-up)
0	5	2	4	67	CABG	Interstitial pneumonia
				75	TAA	***Esophageal varices***
				41	Valve	Cerebral infarction
				70	Valve	***Acute myocardial infarction***
1	2	0	0			
2	9	1	5	46	Valve	Heart failure VAD implantation
				50	Valve	Interstitial pneumonia
				49	Valve	Cerebral infarction
				34	CABG	Acute myocardial infarction
				83	Valve	***Lower limb gangrene***
3	4	1	2	52	Valve	***Pneumonia***
				81	TAA	Acute myocardial infarction
4	5	1	4	65	Valve	***Pneumonia***
				44	Valve	Acute myocardial infarction
				59	TAA	Lower limb gangrene
				48	Valve	Lower limb gangrene
5	1	0	1	55	TAA	Septic Intestinal perforation

Bolded and italicized text represents deceased patients.

CABG = coronary artery bypass grafting, TAA = thoracic aortic surgery, VAD = left ventricular assist device.

## 4. Discussion

In this study, we analyzed the short- and long-term outcomes of patients with SLE undergoing open-heart surgery according to the APS severity classification. There were several significant findings. First, the surgical outcome of open-heart surgery in patients with SLE was good, and there were no hospital deaths, no severe thromboembolic or hemorrhagic complications, and no serious infections. However, perioperative management of patients undergoing these operations was difficult and complex. Second, we focused on blood coagulation abnormalities. We used the APS severity classification, which considers the risk of bleeding and thrombosis, to classify SLE patients before surgery. This severity classification was used to determine the respective perioperative strategies. Third, regarding the follow-up outcomes, there were many rehospitalizations due to cardiovascular events associated with thromboembolism or infections, and careful outpatient follow-up was necessary.

SLE is a chronic systemic autoimmune disease that presents with various clinical manifestations (age of onset, severity, affected organs). In most cases of SLE, the disease is active and persistent. The cardiovascular risk in patients with SLE is 6-fold higher than that in non-SLE patients.^[21–24]^ Schoenfeld et al^[22]^ reviewed 4 cohort studies and 2 case-control studies and reported that there were 1232 coronary artery disease-related events among 15,822 SLE patients, a 2- to 10-fold increased risk of myocardial infarction in SLE patients, and generally a more significant relative risk increase was observed in younger patient groups. Manzi et al^[23]^ compared 498 patients with SLE in a University of Pittsburgh cohort with age-matched controls from the Framingham Heart Study in a retrospective cohort analysis. Young women aged 35 to 44 years with SLE had a very low absolute risk of myocardial infarction; compared with age-matched controls, the relative risk of myocardial infarction was an alarmingly high 52.4-fold (95% confidence interval 21.6–98.5).

It has been reported that the frequency of valvular heart disease, coronary artery disease, and aortic disease increases with the duration of treatment in SLE patients.^[25–32]^ An analysis of SLE patients (n = 5018) and age- and sex-matched controls (n = 25,090) using the Israeli database showed that cardiac valvular disease was more frequent in the SLE group than in the control group (aortic stenosis: 1.08% vs 0.35%, aortic insufficiency: 1.32% vs 0.29%, *P* < .001, mitral stenosis: 0.74% vs 0.21%, *P* < .0001; mitral regurgitation: 1.91% vs 0.39%, *P* < .001). Furthermore, disease prevalence surveys are inadequate in areas with underdeveloped health insurance systems, and valvular disease may be missed even more frequently.^[25]^ Moreover, it has been reported that APS associated with SLE can cause various pathologies and complicate the pathogenesis of valvular heart disease, coronary artery disease, and aortic disease.^[25,29–31]^

In open-heart surgery, the most troublesome issue is blood coagulation abnormalities. Because of bleeding tendency and thromboembolism, systemic complications may directly lead to serious life-threatening events. Coagulation dysfunction such as elevated fibrin degradation products is observed in most patients, especially in those with aortic aneurysms. Therefore, postoperative recovery of platelet count and coagulation function is delayed in patients undergoing aortic surgery, resulting in a more extended hospital stay. In addition, mechanical valves are used in most cases of prosthetic valve replacement, after taking into account the age of SLE patients.^[33]^ Whenever possible, mitral valvuloplasty should be considered as the procedure of choice for mitral regurgitation and mechanical valve replacement for aortic and mitral stenosis. For patients with SLE who have mechanical valves, clinicians should consider permanent and closely monitored warfarin management for those with unstable blood coagulation function. We believe that it is crucial to perform OPCAB without CPB to avoid unstable blood coagulation in CABG and reduce heparin use. By avoiding CPB, we can avoid a decrease in platelet function. However, such perioperative management strategies are inconsistently used, and we believe that strategies for blood coagulation management that can be easily understood and implemented by all team members are desirable.

In open-heart surgery using CPB, the most critical problem is whether the patient has APS complications. It is well known that patients with APS have a higher risk of postoperative bleeding and thrombosis.^[4,31,34–37]^ Therefore, we decided to apply the APS severity classification to all preoperative cases and actively apply the APS management protocol to patients with APS severity of Grade III or higher. In addition, when CAPS is triggered by surgical stress, infection, sepsis, systemic inflammatory response syndrome, or inappropriate treatment, thrombosis and hemorrhage may occur in multiple organs simultaneously, resulting in a high mortality rate.^[4,31,34–37]^ At our hospital, we are currently applying an aggressive APS management protocol for patients with Grade III or higher according to the APS severity classification. For preoperative evaluation, 3 different serum tests are also used to measure antiphospholipid antibodies: the anticardiolipin enzyme-linked immunosorbent assay, LAC test, and anti-beta-2-glycoprotein I enzyme-linked immunosorbent assay. Patients with sustained positive results for all 3 tests are considered as being triple positive and are at a higher risk of thrombosis than patients with single or double-positive results.^[38]^ Fortunately, we did not have any triple-positive cases at our institution; however, some patients have been positive in the past active period. We believe that the same treatment protocol should be used for these triple-positive cases.

We implemented a slightly loosened version of CAPS treatment (glucocorticoids, anticoagulation, intravenous immunoglobulin, and plasma exchange). The original purpose of plasma exchange therapy was to remove autoantibodies, remove other pathogens with large molecular weights, and replenish plasma proteins. Plasma exchange of at least 1.5 times the normal circulating plasma volume should be recommended to achieve these goals. However, in cases such as CAPS, the primary target is the reestablishment of normal coagulation function. We consider that the minimum amount of coagulation factor activity required to expect a physiological hemostatic effect and stability of coagulation function following CPB is approximately 20% to 30% of the normal circulating plasma volume. Therefore, during CPB, only 30% of the total body plasma volume was replaced with fresh frozen plasma. We also recommend administering steroid pulses of 1000 mg methylprednisolone sodium succinate on the day of operation and the first postoperative day. In our case, the patients maintained stable coagulation function without thromboembolism or hemorrhagic complications postoperatively. Furthermore, postoperative platelet recovery was delayed relative to that with OPCAB without CPB; however, the platelet counts recovered within 7 to 10 postoperative days. The most critical problem is postoperative anticoagulation therapy. Control by oral medication, such as warfarin, is unstable and dangerous until the patient’s appetite recovers. Concerning postoperative heparin use, ACT measurement is prone to error in APS patients; thus, measuring activated partial thromboplastin time helped maintain appropriate values.^[39]^

In managing the remote period, a continuation of low-dose aspirin is recommended in high-risk APS cases.^[40]^ In addition, the use of anticoagulants, such as warfarin, is recommended for repeated thromboembolism and deep vein thrombosis.^[41]^ There is a lack of consensus regarding the use of direct oral anticoagulants because of bleeding problems. We avoided direct oral anticoagulants and used warfarin for at least 3 months postoperatively, as postoperative cardiac and renal function was variable and urine output was unstable. We lost 2 patients to thromboembolism due to myocardial infarction or intestinal ischemia and 2 patients to infection. In addition, many patients developed new complications during the follow-up. Nonetheless, the follow-up survival rate was relatively good. Most of the patients had a history of SLE for >30 years, and systemic management, including postoperative anticoagulation, was complicated. Outpatient follow-up was conducted by a team with expertise in collagen medicine, cardiology, and cardiac surgery. We consider this collaboration to be the most valuable treatment for patients with SLE.

There were some limitations to this study. Because this was a retrospective case series of a small number of patients, it was impossible to compare the results with a target group that did not receive any specific treatment in the perioperative period. Also, considering that all participants were patients from a single university hospital, the results of this study may not be generalizable to different populations.

As a long-term complication of SLE, cardiovascular events are a serious life-threatening problem. Cardiovascular surgeons need to develop an individualized treatment protocol that is optimal for each patient, taking into account the risk of bleeding and thrombosis. In the past, many cardiovascular surgeons feared multiple organ failure due to bleeding complications and thromboembolism caused by abnormal blood coagulation. However, we chose to preoperatively assess the severity of these blood clotting-related complications and manage them perioperatively. This protocol allowed us to avoid complications and facilitated perioperative management.

## Acknowledgments

We would like to thank Editage (www.editage.com) for English language editing.

## Author contributions

**Conceptualization:** Tohru Asai, Atsushi Amano.

**Data curation:** Daisuke Endo.

**Formal analysis:** Daisuke Endo.

**Investigation:** Akie Shimada, Shizuyuki Dohi, Kan Kajimoto, Yasutaka Yokoyama, Yuichiro Sato, Yoichiro Machida.

**Methodology:** Satoshi Matsushita.

**Supervision:** Atsushi Amano.

**Writing – review & editing:** Taira Yamamoto.

## Supplementary Material
