# Supplementary material for: Management of cardiovascular surgery in patients with systemic lupus erythematosus including thromboembolism and multiple organ failure prevention: A retrospective observational study
Source: Medicine (Baltimore). 2023 Feb 17;102(7):e32979. doi: 10.1097/MD.0000000000032979 (PMC9936021; doi:10.1097/MD.0000000000032979)
Supplement: Supplementary file 1 [file medi-102-e32979-s001.pdf]

Supplementary Table S1. Preoperative ultrasonic echocardiography and computed tomography

	Isolated CABG	Valvular surgery	Aortic surgery
Left ventricular ejection fraction (%)	56 ± 14	63 ± 11	66 ± 10
Valsalva (mm)	29.8 ± 1.3	30.4 ± 5.3	30.9 ± 2.7
Annulus (mm)	19.2 ± 0.9	21.4 ± 3.8	19.9 ± 2.7
Severe aortic valve stenosis	0	6 (35.3%)	1 (20.0%)
Severe mitral valve regurgitation	0	8 (47.1%)	1 (20.0%)
Severe mitral valve stenosis	0	1 (5.9%)	0
Thoracic aortic aneurysm	0	2 (11.8%)	5 (100%)
Aortic dissection	0	0	2 (40.0%)
Diameter of Ascending aorta (mm)	31.0 ± 3.6	33.2 ± 9.2	36.8 ± 9.1

CABG, coronary artery bypass grafting
