# Supplementary material for: Management of cardiovascular surgery in patients with systemic lupus erythematosus including thromboembolism and multiple organ failure prevention: A retrospective observational study
Source: Medicine (Baltimore). 2023 Feb 17;102(7):e32979. doi: 10.1097/MD.0000000000032979 (PMC9936021; doi:10.1097/MD.0000000000032979)
Supplement: Supplementary file 2 [file medi-102-e32979-s002.pdf]

Supplementary Table S2. Intraoperative patient characteristics and early results

	Isolated CABG	Valvular surgery	Aortic surgery
Emergency surgery	0	2 (11.8%)	1 (20.0%)
Reoperation	1 (25.0%)	0	0
Off-pump CABG	3 (75.0%)	0	0
Conventional CABG	1 (25.0%)	0	1 (20.0%)
Number of arterial grafts	3.5 ± 1.3		
Active infective endocarditis	0	2 (11.8%)	0
Mitral valve plasty	0	6 (35.3%)	1 (20.0%)
Mitral valve replacement	0	3 (17.6%)	0
Aortic valve replacement	0	6 (35.3%)	1 (20.0%)
Aortic root reconstruction	0	2 (11.8%)	0
Total arch replacement	0	0	4 (80.0%)
Hemiarch replacement	0	0	1 (20.0%)
Left atrial appendage amputation	3 (75.0%)	8 (47.1%)	3 (60.0%)
Operative time (min)	291 ± 120	245 ± 74	389 ± 100
Cross-clamp time (min)	0	103 ± 38	127 ± 74
Cardiopulmonary bypass time (min)	213	131 ± 52	205 ± 72
postoperative anticoagulation	1 (25.0%)	9 (52.9%)	2 (40.0%)
Hospital mortality	0	0	0
Low output syndrome	0	0	0
Cerebrovascular accident	0	0	0

Respiratory trouble	0	2 (11.8%)	1 (20.0%)
Infection	0	0	1 (20.0%)
Renal failure	0	2 (11.8%)	1 (20.0%)
Revision for bleeding	0	0	0
New onset of atrial fibrillation	0	5 (29.4%)	4 (80.0%)
ICU stay (days)	1.3 ± 0.5	2.8 ± 1.6	5.4 ± 4.0
Hospital stay (days)	12.8 ± 4.6	15.3 ± 8.9	30.8 ± 9.0

CABG, coronary artery bypass grafting; ICU, intensive care unit
