# Supplementary material for: Management of cardiovascular surgery in patients with systemic lupus erythematosus including thromboembolism and multiple organ failure prevention: A retrospective observational study
Source: Medicine (Baltimore). 2023 Feb 17;102(7):e32979. doi: 10.1097/MD.0000000000032979 (PMC9936021; doi:10.1097/MD.0000000000032979)
Supplement: Supplementary file 3 [file medi-102-e32979-s003.pdf]

Supplementary Table S3. Postoperative blood transfusion and coagulation function

	Isolated CABG	Valvular surgery	Aortic surgery
MAP transfusion	1 (25.0%)	13 (76.5%)	5 (100%)
FFP transfusion	1 (25.0%)	11 (64.7%)	5 (100%)
FFP usage (mL)	240	645 ± 563	1848 ± 429
PC transfusion	0	5 (29.4%)	5 (100%)
Systemic bleeding/stroke trouble	0	0	0
Postoperative AT III	128 ± 8	66 ± 13	69 ± 6
Preoperative platelet ($\times 10^4$/ul)	19.2 ± 3.2	18.4 ± 7.6	17.2 ± 8.7
Postoperative platelet count at POD 1 ($\times 10^4$/ul)	12.1 ± 3.5	11.9 ± 5.6	16.6 ± 5.9
Postoperative platelet count at POD 3 ($\times 10^4$/ul)	14.3 ± 8.1	9.4 ± 4.4	9.2 ± 4.5
Postoperative platelet count at POD 5 ($\times 10^4$/ul)	18.9 ± 9.9	12.4 ± 6.4	9.4 ± 4.3
Postoperative platelet count at POD 7 ($\times 10^4$/ul)	21.6 ± 10.4	16.3 ± 8.1	10.1 ± 4.3
Postoperative platelet count at POD 10 ($\times 10^4$/ul)	24.0 ± 7.1	23.0 ± 9.9	16.3 ± 7.4

AT III, antithrombin III; Postoperative AT III, AT III at the time of admission to the intensive care unit after surgery; CABG, coronary artery bypass graft; MAP, mitral annular plication; FFP, fresh frozen plasma; PC, platelet concentrate; POD, postoperative day
