# Supplementary material for: Management of cardiovascular surgery in patients with systemic lupus erythematosus including thromboembolism and multiple organ failure prevention: A retrospective observational study
Source: Medicine (Baltimore). 2023 Feb 17;102(7):e32979. doi: 10.1097/MD.0000000000032979 (PMC9936021; doi:10.1097/MD.0000000000032979)
Supplement: Supplementary file 4 [file medi-102-e32979-s004.pdf]

**Supplementary Table S4. Blood transfusion and complications according to the severity grades of antiphospholipid antibody syndrome**

| Severity grade of APS | Grade I–II | Grade ≥ III | *P*-value |
|---|---|---|---|
| Number of patients | 16 | 10 | |
| Preoperative hemoglobin (g/dL) | 12.3 ± 1.6 | 9.5 ± 1.8 | .002 |
| Preoperative FDP-P | 2.05 ± 2.25 | 4.51 ± 5.34 | .40 |
| FFP transfusion | 7 (43.8%) | 10 (100.0%) | .004 |
| FFP usage (mL) | 463 ± 592 | 1376 ± 603 | .003 |
| PC transfusion | 4 (25.0%) | 6 (60.0%) | .07 |
| Systemic bleeding/stroke trouble | 0 | 0 | - |
| Preoperative AT III | 108.6 ± 12.8 | 97.7 ± 17.5 | .17 |
| Postoperative AT III | 66 ± 15 | 67 ± 9 | .56 |
| Preoperative platelet count ($\times 10^4$/ul) | 19.5± 7.0 | 16.3 ± 7.2 | .34 |
| Postoperative platelet count at POD 1 ($\times 10^4$/ul) | 12.1 ± 5.7 | 13.9 ± 5.4 | .37 |
| Postoperative platelet count at POD 3 ($\times 10^4$/ul) | 10.8 ± 6.0 | 9.0 ± 3.3 | .50 |
| Postoperative platelet count at POD 5 ($\times 10^4$/ul) | 14.7 ± 8.0 | 9.8 ± 3.7 | .04 |
| Postoperative platelet count at POD 7 ($\times 10^4$/ul) | 18.7 ± 9.1 | 11.6 ± 4.6 | .04 |
| Postoperative platelet count at POD 10 ($\times 10^4$/ul) | 23.5 ± 10.2 | 18.3 ± 6.6 | .26 |
| Postoperative renal failure | 1 (6.3%) | 2 (20.0%) | .29 |
| Respiratory complications | 1 (6.3%) | 2 (20.0%) | .29 |
| Postoperative infection | 1 (6.3) | 0 | .32 |
| Mortality during the follow-up | 3 (18.8%) | 2 (20.0%) | .94 |

AT, antithrombin; FDP-P, fibrin/fibrinogen degradation products; FFP, fresh frozen plasma; PC, platelet concentrate; POD, postoperative day; APS, antiphospholipid antibody syndrome
